# Variability in microcystin quotas during a *Microcystis* bloom in a eutrophic lake

**DOI:** 10.1371/journal.pone.0254967

**Published:** 2021-07-21

**Authors:** Susanna A. Wood, Jonathan Puddick, Ian Hawes, Konstanze Steiner, Daniel R. Dietrich, David P. Hamilton

**Affiliations:** 1 Cawthron Institute, Nelson, New Zealand; 2 Department of Biological Sciences, University of Waikato, Hamilton, New Zealand; 3 Faculty of Biology, University of Konstanz, Konstanz, Germany; 4 Australian Rivers Institute, Griffith University, Brisbane, Australia; INRA/Sorbonne University, FRANCE

## Abstract

*Microcystis* is a bloom-forming genus of cyanobacteria with some genotypes that produce highly toxic microcystin hepatotoxins. In waterbodies where biological and physical factors are relatively homogenous, toxin quotas (the average amount of toxin per cell), at a single point in time, are expected to be relatively constant. In this study we challenged this assumption by investigating the spatial distribution of microcystin quotas at a single point in time on two separate occasions in a lake with a major *Microcystis* bloom. *Microcystis* cell concentrations varied widely across the lake on both sampling occasions (730- and 137-fold) together with microcystin quotas (148- and 362-fold). Cell concentrations and microcystin quotas were strongly positively correlated (R^2^ = 0.89, P < 0.001, n = 28; R^2^ = 0.67, P < 0.001, n = 25). Analysis of *Microcystis* strains using high-throughput sequencing of the 16S-23S rRNA intergenic spacer region showed no relationship between microcystin quota and the relative abundance of specific sequences. Collectively, the results of this study indicate an association between microcystin production and cell density that magnifies the potential for bloom toxicity at elevated cell concentrations.

## Introduction

Eutrophication and climate change have been implicated in a global increase in the frequency and intensity of cyanobacterial blooms [1,2]. Many of the cyanobacteria responsible for these blooms produce toxins that can cause tissue damage from external contact or may be lethal when consumed by humans, livestock, pets and wildlife [3]. Toxic blooms have resulted in major costs to tourism, agriculture, farming and human health worldwide, and loss of ecosystem services and amenities [4].

*Microcystis*, a colony-forming cyanobacterium that produces the eponymous toxin microcystin, forms blooms in most countries around the world and on all continents except Antarctica [5]. More than 250 different congeners of microcystin have been identified [6], all of which act by irreversibly inhibiting eukaryotic serine/threonine protein phosphatases (e.g., 1 and 2a; [7,8]) resulting in hepato-, nephro- and neuro-toxicity [9]. Ingestion of water contaminated with microystins has caused human fatalities [10] and the toxins have been implicated in increased incidences of human liver cancer in some countries [11–16].

Not all *Microcystis* strains are capable of toxin production, and a single species can contain both toxic and non-toxic genotypes. In lakes, microcystin concentrations are largely regulated by the abundance of toxic genotypes, which can shift over the duration of a bloom and vary spatially, resulting in correlations between cell abundance with toxin concentration [17,18]. Given the reliance of many monitoring programmes on cell counts rather than toxin detection, an understanding of the variables that most affect toxin production is beneficial. Research on *Microcystis* spp. *in vitro* has shown correlations between microcystin quotas (total intracellular microcystins per cell) and a range physiochemical parameters, including nutrients [19–21] and temperature [22], and other physiological variables such as growth stage [23,24]. However, laboratory-based studies are often contradictory and usually only induce changes in microcystin quotas by three- or four-fold. In addition, the factors that up- or down-regulate production *in vitro* [19,20,25] may poorly reflect *in situ* conditions. This is because, the highly controlled laboratory growth conditions do not necessarily mimic what happens in the environment, and changes can occur in cyanobacteria maintained in culture for extended periods, such as loss of colonial morphology [26,27].

Increasingly, studies have focused on microcystin regulation *in situ*, in particular through the incorporation of molecular techniques such as metatranscriptomics into field ecological studies, e.g., [28]. However, most studies that have measured microcystins *in situ* track the toxin at one location over long temporal scales, or if they have focused on spatial variability they have assessed total microcystins rather than determining microcystin quotas [29,30]. Examining microcystin quotas, rather than total microcystins, may allow greater insight into the variables that regulate production of the toxin. Using field surveys, Wood et al. (2010 and 2012) [31,32] reported changes in microcystin quotas of nearly 20-fold over 5 h and microcystin-E-synthetase (*mcyE*) gene expression of >400-fold as *Microcystis* cell concentrations increased during formation of a bloom. Both of these studies tracked microcystin production during bloom formation and provided no information on how microcystin quotas varied instantaneously across a lake.

The aim of this study was to investigate the spatial distribution of microcystin quotas on two separate occasions in a lake which had a major *Microcystis* bloom. We hypothesized that microcystin quotas would not be constant but would be correlated with total *Microcystis* cell concentrations. To address this hypothesis, samples were collected from a highly eutrophic lake on two occasions, twelve months apart. A combination of microscopy, quantitative PCR (QPCR) and liquid chromatography-tandem mass spectrometry (LC-MS/MS) was used to determine microcystin quotas in each sample. The abundance of *Microcystis* genotypes was also assessed, by high-throughput sequencing (HTS) of the intergenic spacer (ITS) region in a selection of samples, to examine the influence of genotype composition on microcystin quotas.

## Materials and methods

### Spatial distribution of microcystin quotas

**Fig 1:**
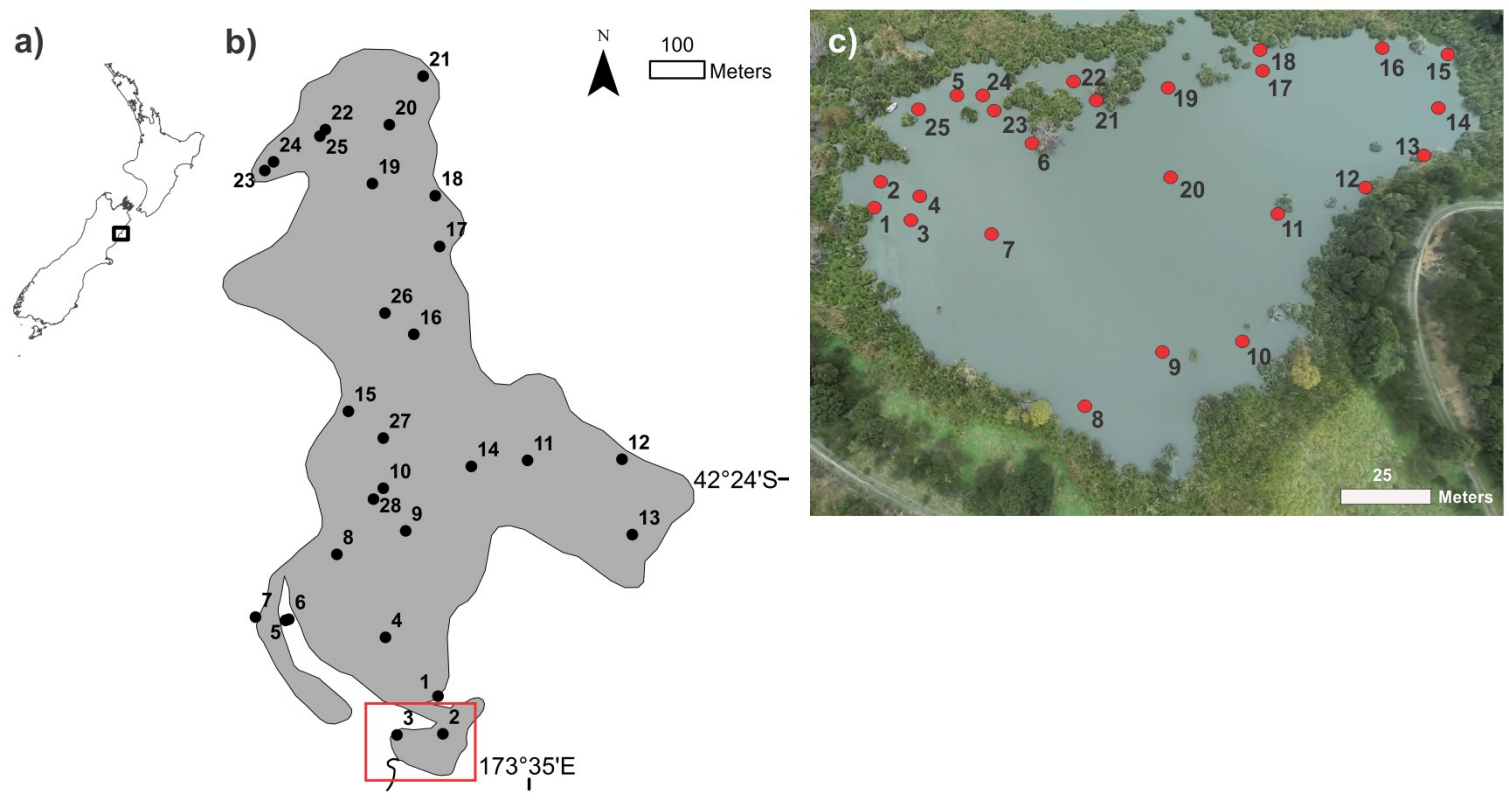
Location of sampling sites in Lake Rotorua. a) New Zealand map showing location of Lake Rotorua, b) 28 lake-wide stations (shape files from www.arcgis.com), and c) 25 bay stations. Red box in b) shows location of the bay study. The image used in (c) was taken using a drone.

Lake Rotorua (42°24’05S, 173°34’57E) is a small (0.55 km^2^), shallow (max. depth 3 m), eutrophic lake in northeast South Island, New Zealand [33]. Water samples were collected between 1400 to 1530 h on 23 April 2013 at 28 stations distributed across the lake and between 1520 to 1640 h on 15 April 2014 at 25 stations in a small partially enclosed bay at the southern end of the lake (Fig 1). The southern bay was sampled to increase the spatial resolution of sampling microcystin quotas as *Microcystis* scums had been observed to be highly variable spatially and temporally in this area. During the lake-wide study sampling was designed to allow for collection of an approximately equal number of mid-lake (n = 11) and littoral zone (n = 9), in addition to targeting areas where scum formation was visible (n = 6). During the bay study, rather than targeting ‘zones’ we specifically selected areas where the surface cell concentrations were visibly different. Surface water samples (upper 5 mm) were collected by partially submerging a Falcon tube (50 mL) horizontally just below the water surface to allow the water to run into the tube until it was at least three quarters full.

Samples were processed immediately. One set of sub-samples (1.5 mL) was preserved using Lugol’s iodine for *Microcystis* cell enumeration. Another set of sub-samples (1–3 mL; unfiltered) was frozen immediately in liquid nitrogen for total microcystin analysis. A final set of sub-samples (3–19 mL) for DNA analysis was collected on Whatman GF/C glass microfiber filters which were placed in a sterile Eppendorf tube and stored on ice. The first 0.8 mL from the DNA filtrate was collected directly in a glass autosampler vial for extracellular microcystin analysis and stored on ice. All processed samples were stored at −20 °C within 3–4 h of field processing except for those for cell enumeration, which were stored in the dark at ambient temperature.

During the bay study, temperature, pH, turbidity, conductivity and dissolved oxygen (DO) were measured just below the surface (≤3 cm) using an EXO2 water quality SONDE (Yellow Spring Instruments, Ohio, USA).

### Laboratory analysis

*Microcystis* enumeration was undertaken using an inverted microscope (Olympus CKX41, Wellington, New Zealand). Samples were lightly ground mechanically (Wheaton Tissue Grinder, Wheaton, NJ, USA) for about 30 s to break up *Microcystis* colonies and allow enumeration of individual cells [34]. Ground subsamples (0.1–1 mL) were settled in Utermöhl chambers [35], and *Microcystis* cells from 10 random fields were counted at 400× magnification.

A standard curve for the *mcyE* QPCR assay was prepared using a culture of *Microcystis* CAWBG617 (isolated from Lake Rotorua [36]). The culture was grown in a glass flask (500 mL) in MLA medium [37] under a light regime of 90 μmol m^-2^ s^-1^ with a 12 h:12 h light:dark cycle, at a temperature of 18 °C ± 1 °C. A subsample (60 mL) of the culture in an expontential growth phase was filtered onto a glass fibre filter (Whatman GF/C) and the filter was stored frozen (−20 °C) for DNA extraction. A second subsample (10 mL) was preserved using Lugol’s iodine and the cells were enumerated microscopically as described above.

DNA was extracted from the GF/C filters (for both the study samples and the QPCR standard) using a DNeasy PowerSoil Kit (Qiagen, USA) according to the protocol supplied by the manufacturer. All DNA samples were screened in duplicate for inhibition using an internal control assay. Each 12.5 μL reaction volume contained 6.25 μL KAPA Probe Fast QPCR Kit Master Mix (2×), 1 μL of primers targeting the internal transcribed spacer region 2 of the rRNA gene operon of *Oncorhynchus keta* salmon sperm (0.4 μM, Sketa F2 and Sketa R3; IDT, USA [38]), 1 μL TaqMan probe synthesised with a FAM reporter dye at the 5´end and a Black Hole Quencher 2 at the 3´end (0.2 μM; Sketa P2; IDT, USA [38]), 1 μL extracted salmon sperm DNA (15 ng; Sigma, USA) and 1 μL of template DNA. The cycling profile was 95 °C for 3 min, followed by 50 cycles of 95 °C for 3 s and 58 °C for 10 s. When inhibition was observed, samples were diluted (1/10) with Milli-Q water and re-analysed.

Quantitative-PCR was used to enumerate the copy numbers of the *mcyE* gene. Samples were anlaysed in triplicate in 12.5 μL of reaction mix containing 6.25 μL KAPA Probe Fast qPCR Kit Master Mix (2×), 1 μL of primers targeting a region within the *mcyE* open reading frame of the microcystin synthase gene (0.4 μM, mcyE-F2 and MicmcyE-R8 [39]), 0.2 μL of mcyE probe [40] and 1 μL of template DNA. QPCR efficiency was >0.8. DNA from CAWBG617 was used to generate a five-point linear standard curve (R^2^ >0.99) ranging from 10.8×10^6^ to 10.8×10^2^ cells mL^-1^.

Twelve samples from the bay study (encompassing a range of microcystin quotas) were selected for HTS analysis of the ITS region between the 16S and 23S rRNA genes. A region of approximately 500 bp was amplified using cyanobacterial-specific primers; ULR [41,42] and CSIF [43], modified to include Ilumina^™^ adapters. PCR reactions were performed in 50-μL volumes containing 25 μL of AmpliTaq Gold^®^ 360 Master Mix (Life Technologies), 5 μL CG inhibitor (Life Technologies), 0.5 μM of each primer, and template DNA (ca. 20 ng). PCR cycling conditions were: 95 °C for 10 min, followed by 27 cycles of 95 °C for 30 s, 50 °C for 45 s, 72 °C for 45 s, and a final extension of 72 °C for 7 min. PCR products were visualized with 1% agarose gel electrophoresis with Red Safe DNA Loading Dye and UV illumination. PCR products were purified (Agencourt^®^ AMPure^®^ XP Kit; Beckman Coulter, USA), quantified (Qubit^®^ 20 Fluorometer, Invitrogen), diluted to 10 ng μL^-1^ and submitted to New Zealand Genomics Limited (Auckland, New Zealand) for library preparation. Libraries were sequenced on a MiSeq Illumina^™^ platform (2 × 300 reads).

Overlapped raw sequence reads were denoised, trimmed and filtered prior to downstream analyses. Paired-end reads were assembled into contigs using USEARCH [44]. Merged reads of <200 bp were discarded. The data were then filtered with VSEARCH [45], and reads with more than one expected error per read were discarded [46]. The data were dereplicated by removing all non-unique sequences, to make downstream computation faster. Operational taxonomic units (OTUs) were generated using VSEARCH by clustering each unique sequence at the 99% identity threshold. Non-unique reads were then mapped back onto these clusters, and any cluster that contained fewer than 10 sequences was discarded. Taxonomy was assigned to each OTU using a reference database which was constructed using cyanobacterial ITS sequences from GenBank [47]. Only ITS sequences assigned to *Microcystis* were utilized for further analysis.

Subsamples collected for extracellular microcystin required no further preparation prior to analysis. Subsamples for ‘total’ microcystin analysis were freeze-thawed and sonicated (30 min, 60 kHz) four times after adding formic acid (final concentration 0.1% v/v). The extract was clarified by centrifugation (10,000 × *g*, 5 min) and the supernatant was placed in a glass autosampler vial. The extraction efficiency of this aqueous microcystin extraction was previously validated using comparison to that extracted in methanol [48].

Microcystin samples were analyzed directly or diluted (1/10 to 1/100 with 50% methanol containing 0.1% formic acid) by LC-MS/MS multiple-reaction monitoring as described in Puddick et al. (2016) [49]. Compounds were separated on an Acquity I-Class ultra-performance liquid chromatography system (Waters Co.) using a C_18_ column (Waters Acquity BEH-C_18_, 1.7-μm, 50×2.1 mm) maintained at 40 °C in a column oven. Sample components were eluted using a flow rate of 0.4 mL min^-1^ and a gradient of 10% acetonitrile (mobile phase A) to 90% acetonitrile (mobile phase B), each containing 100 mM formic acid and 4 mM ammonia. The samples were injected at 5% B and held for 12 s before a linear gradient up to 35% B over 24 s, to 50% B over a further 72 s and to 65% B over a final 42 s, before flushing with 100% B and returning to the initial column conditions. Sample components were analyzed on a Xevo-TQS mass spectrometer (Waters Co.) operated in positive-ion electrospray ionisation mode (source temperature 150 °C; capillary voltage 1.5 kV; nitrogen desolvation gas 1,000 L hr^-1^ at 500 °C; cone gas 150 L hr^-1^). Multiple-reaction monitoring channels for each microcystin congener and nodularin-R assessed for the *m/z* 135 fragment ion produced from the protonated molecular cations of each toxin ([M+2H]^2+^ for MC-RR and variants; [M+H]^+^ for the others).

Retention times for microcystins were within ±0.02 min compared to a quality control material produced from microcystin/nodularin standards and an extract of a well-characterised cyanobacteria strain (*Microcystis* CAWBG11; [50]). A five-point mixed external calibration curve was produced using the microcystin congeners; MC-RR, MC-YR and MC-LR (DHI Lab Products, Denmark). The limit of detection for MC-RR, MC-YR, MC-LR and nodularin in ‘total’ and extracellular microcystin samples was 0.02 ng mL^-1^ and the limit of quantitation was 0.06 ng mL^-1^. Matrix effects were assessed in a selection of lake water samples by fortifying an aliquot of sample with the quality control material described above. No compensation for suppression/enhancement effects was made as the results were within ±10% of the expected result.

Microcystin quotas were calculated as the amount of microcystin per microcystin-producing cells (determined using *mcyE* QPCR). The concentration of all microcystin congeners observed in the samples was summed, deducting the dilution-adjusted extracellular microcystin concentration from the dilution-adjusted ‘total’ microcystin concentration, and divided by the concentration of microcystin-producing *Microcystis*.

Visualization of *Microcystis* cell densities and microcystin quotas for each study (lake-wide and bay) were spatially interpolated across a rectangular surface then clipped to the area of the lake before generating lake and bay-wide plots in ArcGIS 10.1 (Esri, CA, USA).

Statistical analyses were conducted using R software [51]. Two-parameter linear regression was used to determine relationships between: *Microcystis* cell concentration and microcystin quotas; measured physiochemical variables and microcystin quotas (bay study only); and the abundance of the top five ITS OTUs and microcystin quotas (only selected samples from the bay study).

Principal component analysis (PCA) was performed in R [51] to determine which predictors measured in the bay study (*Microcystis* cell concentration, turbidity, conductivity, water temperature, pH and DO) held the most explanatory power. The explanatory power of each principal component (PC) was evaluated using Kaiser’s criterion (threshold of variance > 1), scree plots and biplots with datapoints coded using the microcystin quota value. Eigenvalues and eigenvectors for the retained PCs were also visualised using biplots with arrows expressing the direction and strength (represented by the arrow length) of each predictor. A rotation matrix was used to correlate the contribution of predictor variables to each PC and identify predictor variables with low explantory power (eigenvalues between −0.4 and 0.4). Highly correlated predictors were identified using Pearson’s correlation (r > 0.8) and highly co-linear predictors were assessed by their variance inflation factor (VIF; > 3). When highly correlated and co-linear predictors were identified, the strongest predictor (as evaluated in the PCA) was retained. Multiple linear regression analysis was undertaken in R [51] using the predictor variables retained following PCA and data simplification.

## Results

### Whole lake and bay study

**Fig 2:**
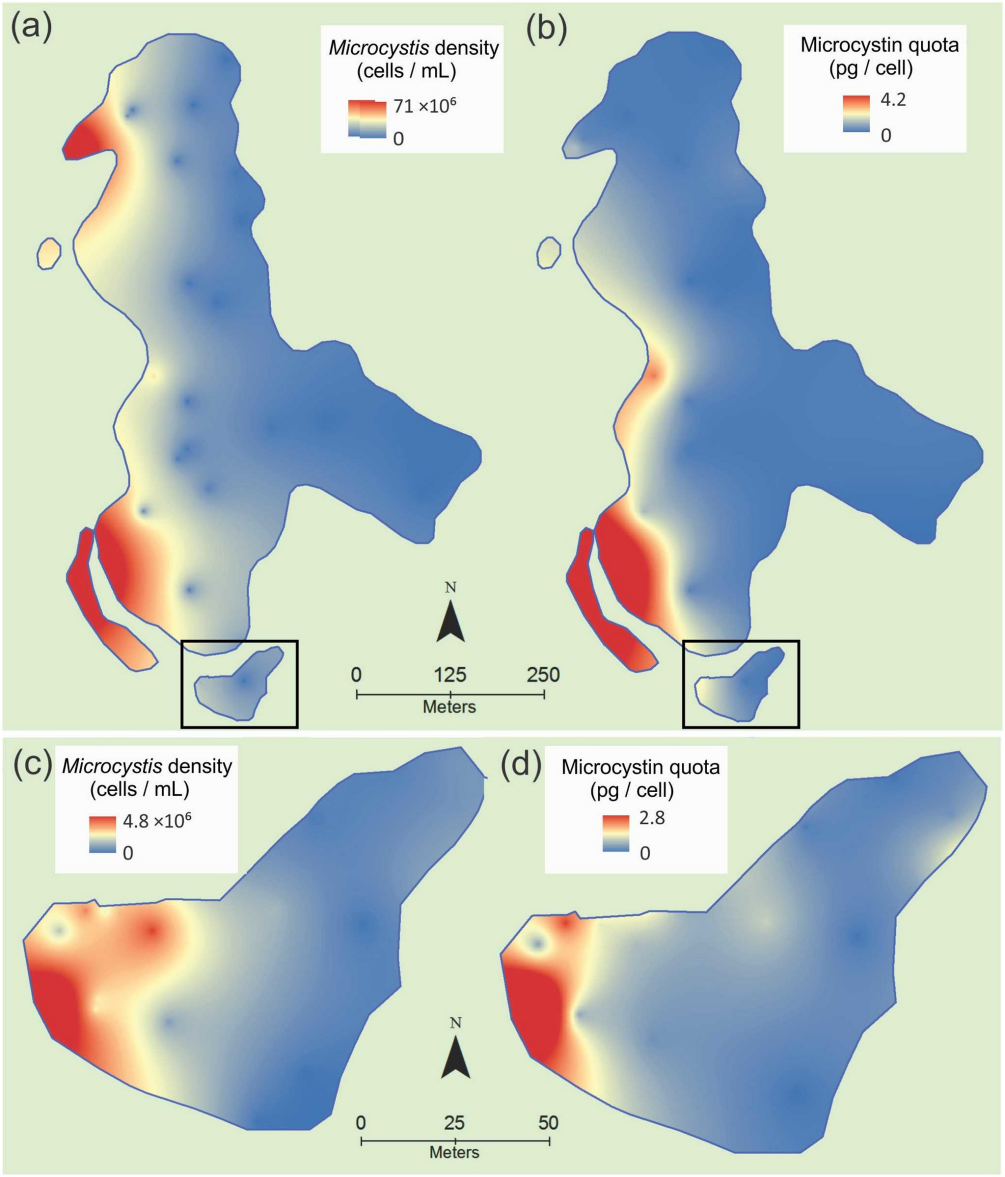
*Microcystis* cell density (a and c) and microcystin quotas (b and d) in Lake Rotorua (Kaikoura, New Zealand) across the whole lake (a and b, 23 April 2013, 1400–1530 h) and the southern bay (c and d, 15 April 2014, 1520–1640 h). The southern bay is shown in (a) and (b) surrounded by a black box.

*Microcystis* cell concentrations varied 730- and 140-fold in the lake-wide and bay surveys, respectively, with the highest densities (71 × 10^6^ and 4.8 × 10^6^ cells mL^-1^ respectively) recorded on the western sides of the lake and the bay (Fig 2a and 2c). Total microcystin concentrations varied 14,300- and 12,500-fold across the lake and bay respectively (min. 0.0003 and 0.03 μg L^-1^, max. 4.3 and 376 μg L^-1^). Microcystin quotas varied 148- and 362-fold across the lake and bay respectively (Fig 2b and 2d; min. 0.03 and 0.008 pg cell^-1^, max. 4.2 and 2.8 pg cell^-1^), and in a similar pattern to the cell concentrations. The percentage of toxic genotypes did not vary greatly across the sample sets and was relatively low, ranging from 1 to 7% in the lake study, and from 2 to 32% in the bay study. In the bay, total *Microcystis* cell density was positively linearly related to the total microcystin concentration (R^2^ = 0.68, P < 0.001) and microcystin quota (R^2^ = 0.67, P < 0.001; Supplementary Information S1 in S1 File). In the whole lake, the relationship between *Microcystis* cell concentration and total microcystin concentration was also strong and significant (R^2^ = 0.91, P < 0.001; Supplementary Information S2a in S1 File), but the relationship between cell concentration and microcystin quotas was weaker than observed in the bay (R^2^ = 0.29, P = 0.003; Supplementary Information S2b in S1 File). The weaker relationship for the lake was largely due to two samples in bays in the northwest of the lake (samples 23 and 24) and one sample in the southwest (sample 7) with low microcystin quotas but high cell densities (Fig 2a and 2b). Removing these samples substanitally improved the microcystin quota–cell density relationship (R^2^ = 0.89, P < 0.001; Supplementary Information S2d in S1 File). Relationships between microcystin quotas and measured physiochemical variables (temperature, pH, turbidity, conductivity and DO; bay study only) were weak (R^2^ ≤ 0.24) but were significant for conductivity (P = 0.04), and temperature (P = 0.01; Supplementary Information S3 in S1 File).

**Fig 3:**
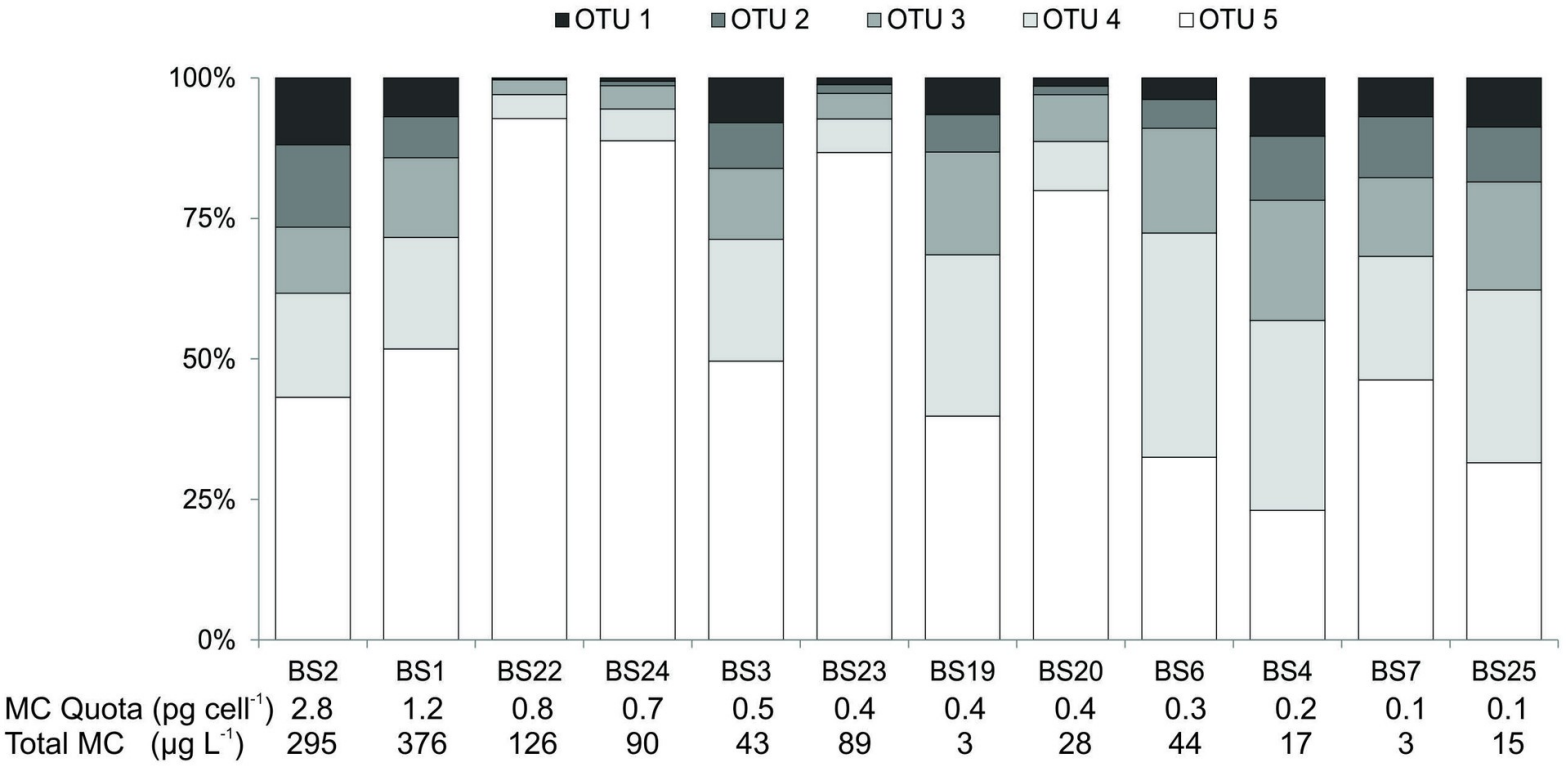
Stacked column graph representing the relative distribution of different operational taxonomic units (OTUs) from *Microcystis* intergenic spacer regions in twelve samples from the bay study (see Fig 1c for location of samples) ordered by decreasing microcystin quota.

The *Microcystis* ITS genotype composition of twelve samples collected from the bay study was assessed by HTS to determine if the relative abundance of genotypes (with potentially differing microcystin production capacities) may have influenced the observed microcystin quotas. Nineteen OTUs were identified in the *Microcystis* ITS analysis, of which five contributed 60%, 15%, 10%, 5% and 4% of the total sequence reads over all samples. The relative abundance of these five genotypes varied between samples, but OTU5 was dominant in all but two of the samples (Fig 3). Linear regression analysis of the microcystin quotas and the relative abundance of the five major OTUs showed weak and nonsignificant relationships (R^2^ ≤ 0.11, P ≥ 0.3; Supplementary Information S4 in S1 File).

The influence of *Microcystis* cell concentrations and physiochemical parameters (temperature, pH, turbidity, conductivity and DO) on microcystin quotas for the bay study samples was evaluated using PCA. Three PCs were required to describe a reasonable proportion of the variance observed in the dataset (81%; Supplementary Information S5a in S1 File) and the addition of more PCs would not have added substantial explanatory power (tailing off observed in the scree plot; Supplementary Information S5b in S1 File). Biplots for the different combinations of the PCs suggested that all three PCs demonstrated some potential for defining the microcystin quotas of the samples, with groupings of microcystin quotas observed for PC1 vs. PC2 and PC1 vs PC3 (Supplementary Information S5c in S1 File). Correlations between each PC and the original data showed that PC1 was driven by *Microcystis* cell concentration, turbidity, water temperature and conductivity, PC2 was driven by DO, and PC3 was driven by turbidity and pH (Supplementary Information S5d in S1 File). Different biplot vectors of the four PC1 predictors indicated that each should be retained in the analysis (Supplementary Information S5e in S1 File). This was reinforced by low Pearson’s correlations between each predictor (R ≤ 0.65; Supplementary Information S5f in S1 File) and low VIFs, indicating that the predictors were not co-linear (≤ 2.7; Supplementary Information S5g in S1 File).

Multiple linear regression of microcystin quotas using all predictor variables (*Microcystis* cell concentration, temperature, turbidity, conductivity, dissolved oxygen and pH) yielded a model with a multiple-R^2^ of 0.71 and an adjusted-R^2^ = 0.62 (p < 0.001; Supplementary Information S6a in S1 File). As the regression relationship between microcystin quotas and *Microcystis* cell concentrations had a R^2^ of 0.67 and an adjusted-R^2^ of 0.65 (p < 0.001; Supplementary Information S1b and S6b in S1 File), cell concentrations provided the majority of the predictive power in the model and the inclusion of the physiochemical parameters only marginally improved the performance of the model.

## Discussion

### Variability in microcystins quotas

Our results indicate that *Microcystis* cell concentrations positively influenced microcystin quotas. This aligns similarly with positive relationships from observations of *Microcystis* scums at Lake Rotorua [31,32] and a mesocosm study where water column samples were collected at fine resolution [49]. In a recent labortory study, Wang *et al*. (2021) [52] also observed upregulation in *mcy* expression and microcystin concentrations when cell density reached approx. 22 × 10^6^ cells mL^-1^. Increases in acyl-homoserine lactones occurred concurrently, leading the authors to suggest a role for microcystins in quorum sensing.

Sequencing of the ITS has previously been used to evaluate changes in populations of *Microcystis* genotypes, as the gene is subject to high levels of sequence variation [53,54]. In the samples assessed from the bay study, relationships between OTU relative abundance and microcystin quotas were not significant, indicating that *Microcystis* genotype composition was not a major contributing factor to microcystin quotas in the samples. Despite the samples being collected from a relatively small area and at approximately the same time, a range of genotype compositions was observed in the assessed samples. OTU5 was the dominant genotype in the majority of the samples and exhibited the largest variation in relative abundance (min. 21% and max. 90%). This differs from a study of the genotype composition of postively-buoyant *Microcystis* colonies from the same study lake (Lake Rotorua, Kaikoura), where low levels of genotype variability were observed with one genotype comprisinig the majority of the sequence reads (72% in one samples and ≥90% in the other samples; note that OTUs from the colony study do not match those from the present study) [55]. This suggests that the sampling method used in the present study captured a wider array of the *Microcystis* genotypes present in the lake than the earlier study that targeted large *Microcystis* colonies and/or that the genotype composition within a lake can vary markedly between years.

Inclusion of all data points from the lake-wide study in the analysis produced weak relationships between *Microcystis* cell density and microcystin quotas. This was largely due to two samples collected from a bay at the north-west end of the lake. A dense surface scum in this bay had persisted for several days, possibly enhanced by entrapment of oxygen bubbles in extracellular polymeric substances that increase buoyancy [56]. This ‘stagnant’ *Microcystis* scum was in stark contrast to the transient scums in other regions of the lake where wind movement, water currents and cellular buoyancy likely contributed to the variability. Further research is required to determine the reason for the low microcystin quotas in ‘stagnant’ samples.

During the bay study, physicochemical parameters were measured at each sampling site using the SONDE probe. Conductivity and temperature had a weak but significant linear regression relationship with microcystin quota, with PCA indicating that all of the physiochemical predictor variables (alongside *Microcystis* cell concentration) provided some explanatory power. However, multiple linear regression analysis indicated that the physiochemcial variables only had a low level of predictive power; whilst *Microcystis* cell concentrations explained most of the variation in microcystin quotas.

In contrast to the results of the present study, the relationship between *Planktothrix agardhii* biomass and microcystin quotas in four waterbodies in Italy showed limited variability in microcystin quotas [57]. This observation might indicate that microcystins have different ecological roles among cyanobacterial species or that they are up-/down-regulated by other factors. Additionally, the *Planktothrix* in the Italian study did not form surface scums, with samples taken from depths of 1–5 m and 10 m to coincide with the subsurface layer where it concentrated, although surface scums have been reported in some instances [58]). The different sampling methodologies and distributions of the *Microcystis* and *Planktothrix* studies limits the ability for comparative assessment of changes in quotas.

## Conclusions

The data in our study provide compelling evidence of an association between increased microcystin production and high cell densities in *Microcystis*. The combination of high cell concentrations in blooms with elevated cell microcystin quotas has important implications for magnifying the toxin content of blooms. The reasons for the upregulation remain unknown but lend support to hypotheses such as cell-to-cell signaling, gene regulation, or as a reponse to oxidative stress and photodamage [25,59,60]. Further studies which aim to characterise the micro-environment in a scum, in concert with the use of molecular approaches such as metatranscriptomics, could provide insights into the up- and down-regulation of microcystins, their ecological role and chemical ecology.

## Supporting information

S1 File Additional statistical analysis results. (DOCX)
