# Supplementary material for: Variability in microcystin quotas during a Microcystis bloom in a eutrophic lake
Source: PLoS One. 2021 Jul 21;16(7):e0254967. doi: 10.1371/journal.pone.0254967 (PMC8294494; doi:10.1371/journal.pone.0254967)
Supplement: S1 File — (DOCX) [file pone.0254967.s001.docx]

Supplementary Information for:

Variability in microcystin quotas during a *Microcystis* bloom in a eutrophic lake

**Supplementary Information S1:** Scatter plots of the relationship between *Microcystis* cell concentrations and **a)** total microcystin concentration and **b)** microcystin quotas from the ‘bay study’.


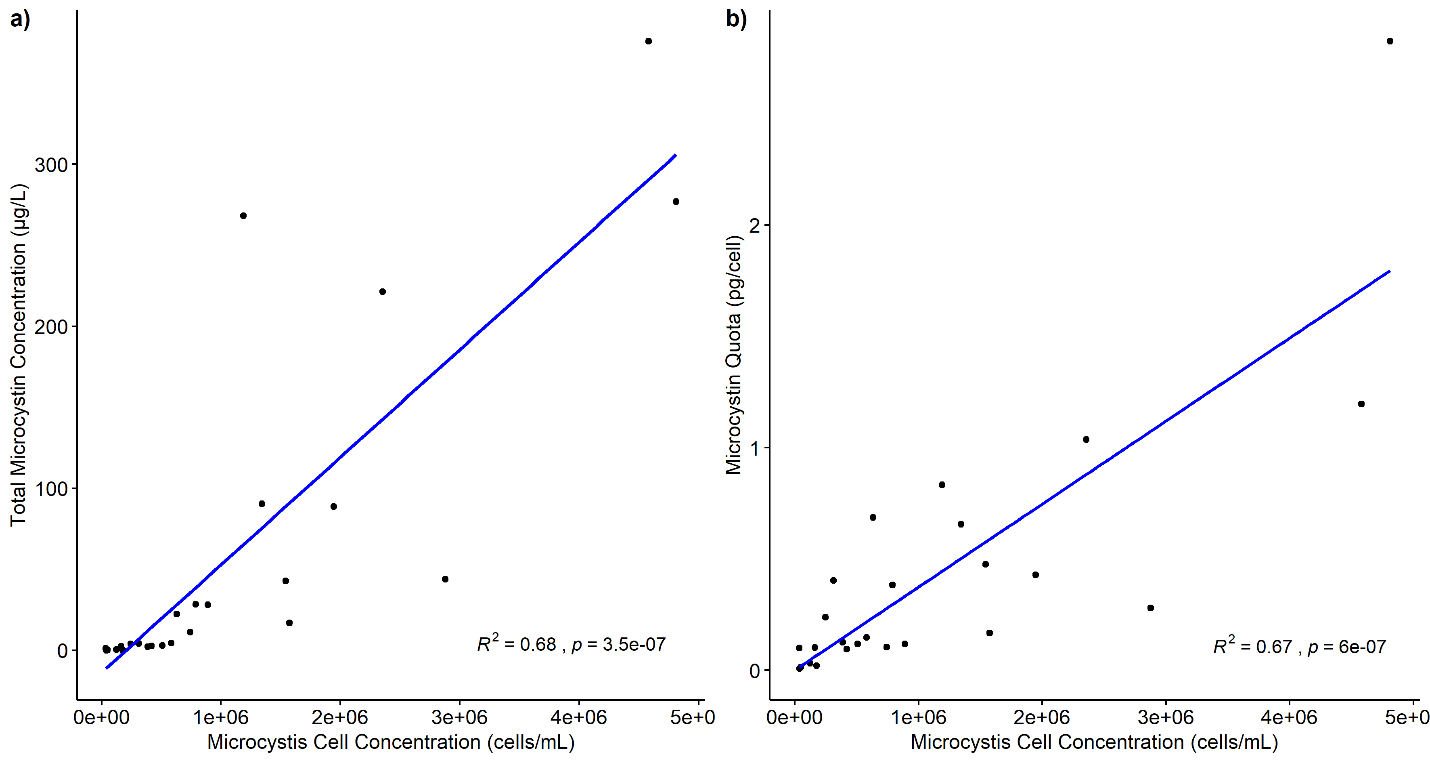


**Supplementary Information S2:** Scatter plots of the relationship between *Microcystis* cell concentrations and total microcystin concentration (**a** and **c**) and microcystin quotas (**b** and **d**) from the ‘whole lake study’ using all data (**a** and **b**) and with outliers from northwest/southwest bays of Lake Rotorua removed (**c** and **d**).


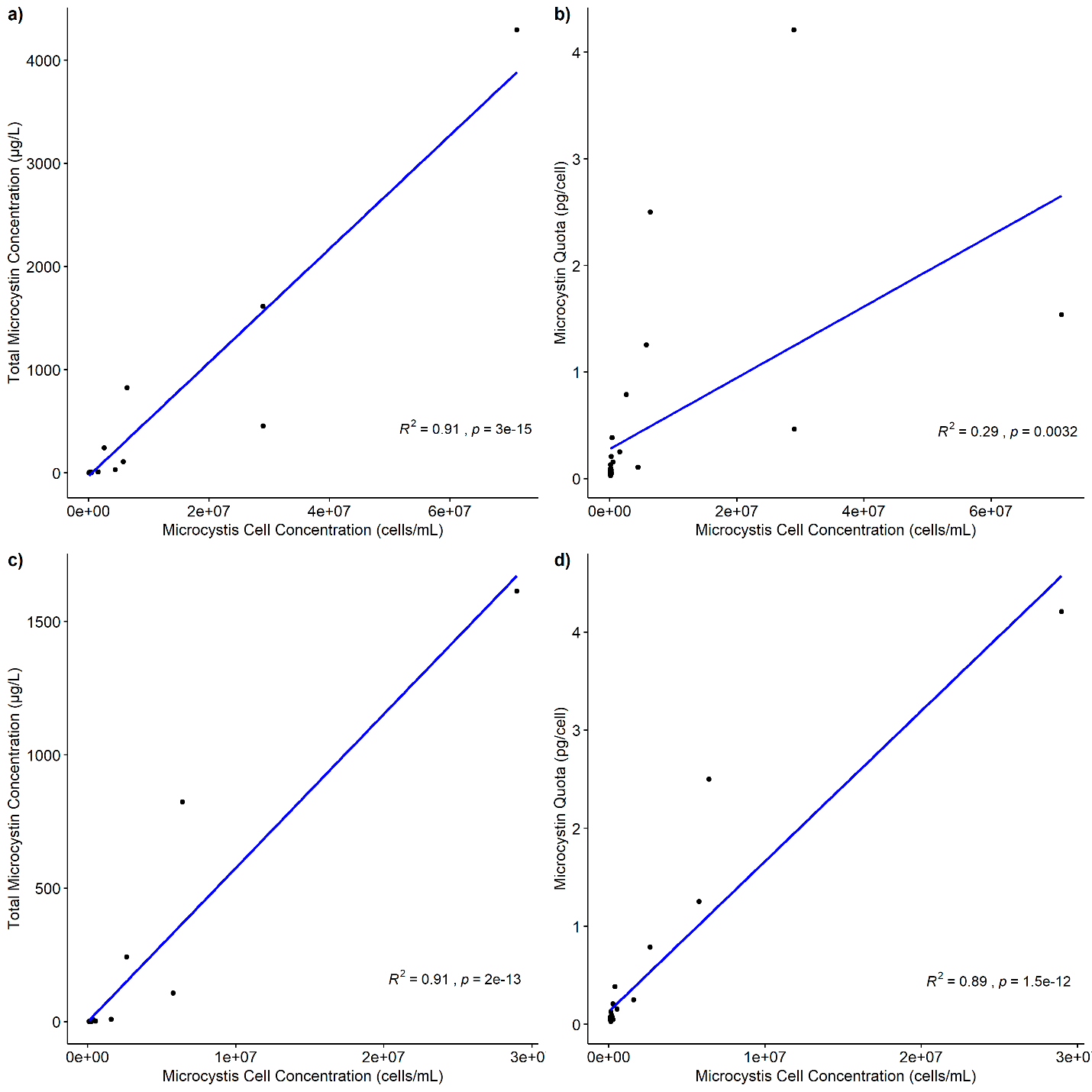


**Supplementary Information S3:** Scatter plots of the relationship between physiochemical measurements and microcystin quotas from the ‘bay study’; **a)** water temperature, **b)** turbidity, **c)** conductivity, **d)** pH, and **e)** dissolved oxygen.


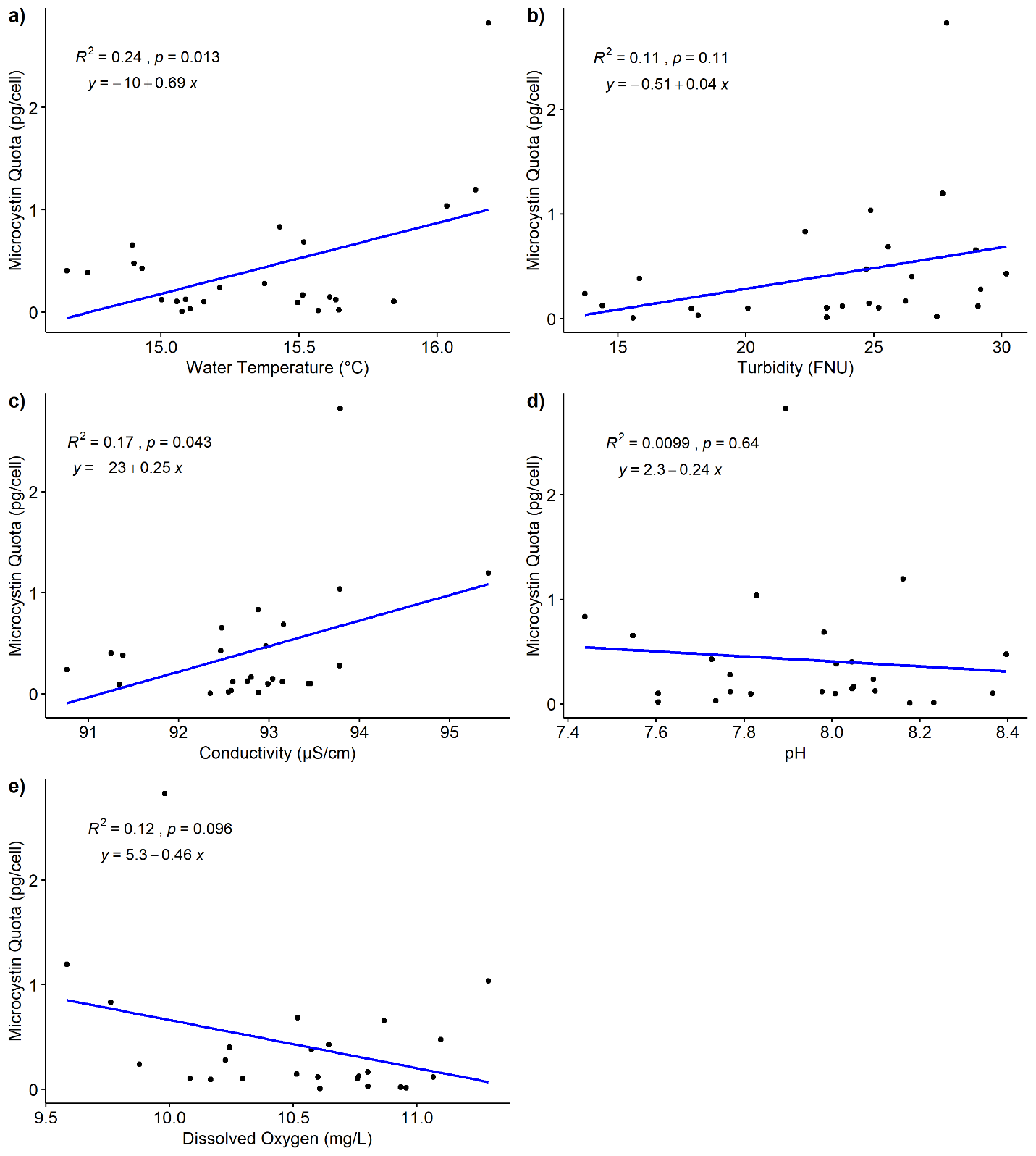


**Supplementary Information S4:** Scatter plots of the relationship between the relative abundance of operational taxonomic units (OTUs) for the *Microcystis* 16S-23S rRNA intergenic spacer region; **a)** OTU5, **b)** OTU4, **c)** OTU3, **d)** OTU2, and **e)** OTU1.


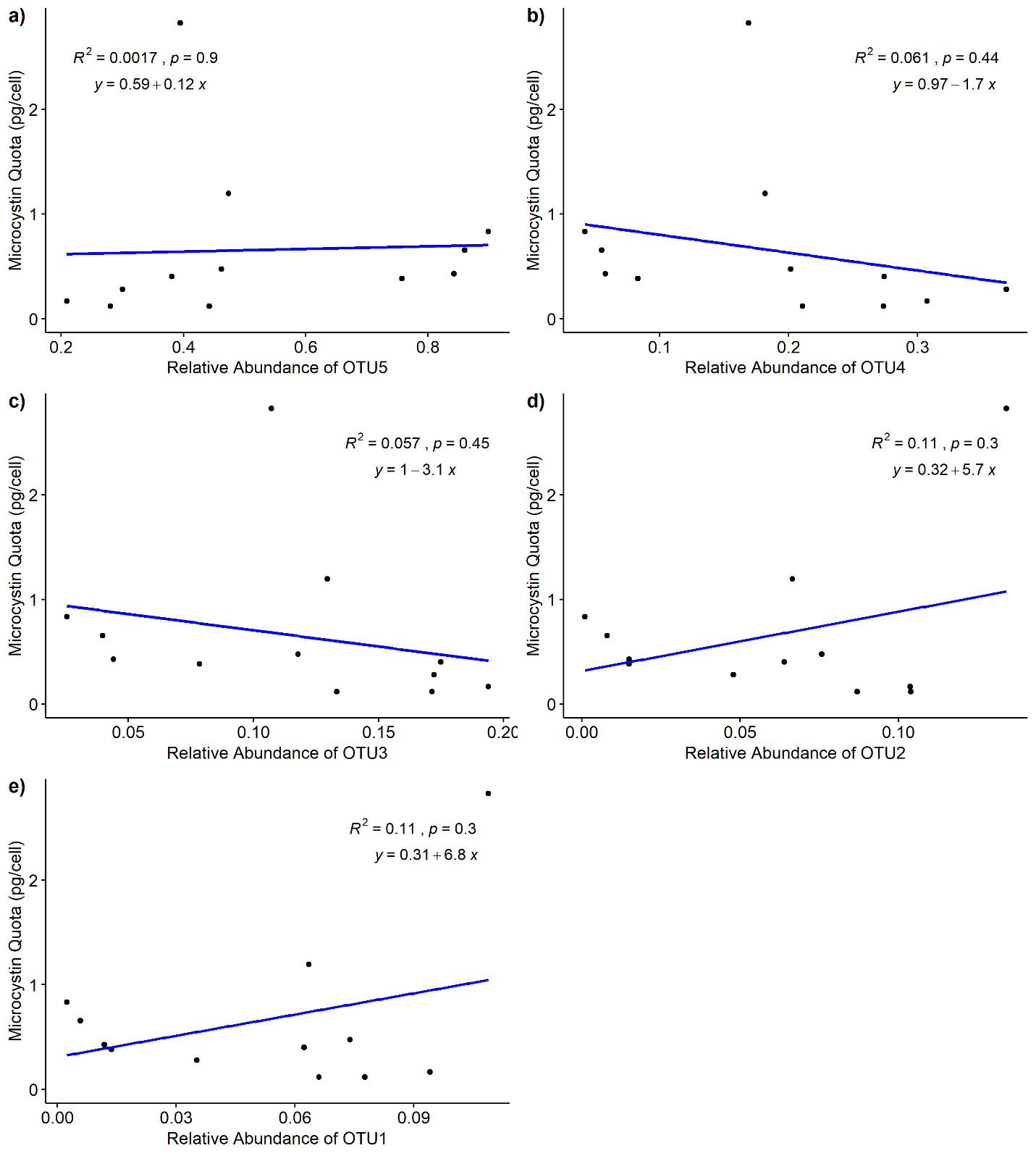


**Supplementary Information S5:** Principal component analysis (PCA) and data simplification for multiple linear regression analysis to assess the influence of cell concentration and physiochemical predictors on microcystin quotas in the ‘bay study’ samples.

**S5a:** Cumulative proportion of the variance for principal components (PCs; emboldened values indicate the PCs required to account for ≥ 80% of the variance).

**S5b:** Scree plot for assessing the strength of PCs.


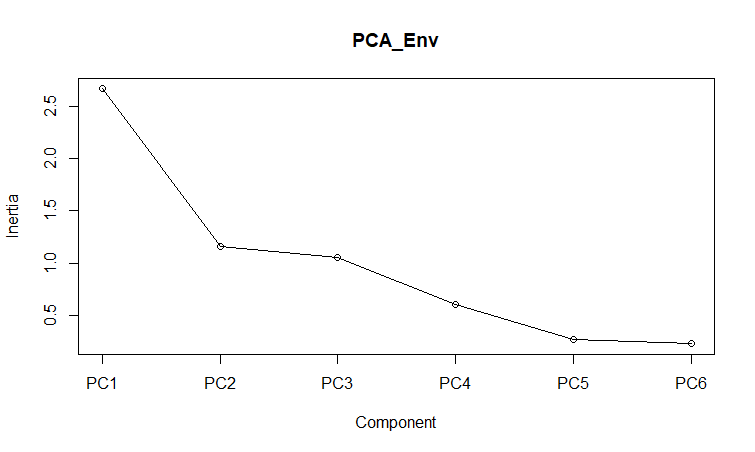


**S5c:** Biplots for combinations of PC1, PC2 and PC3 with datapoints colour-coded by the microcystin quota.


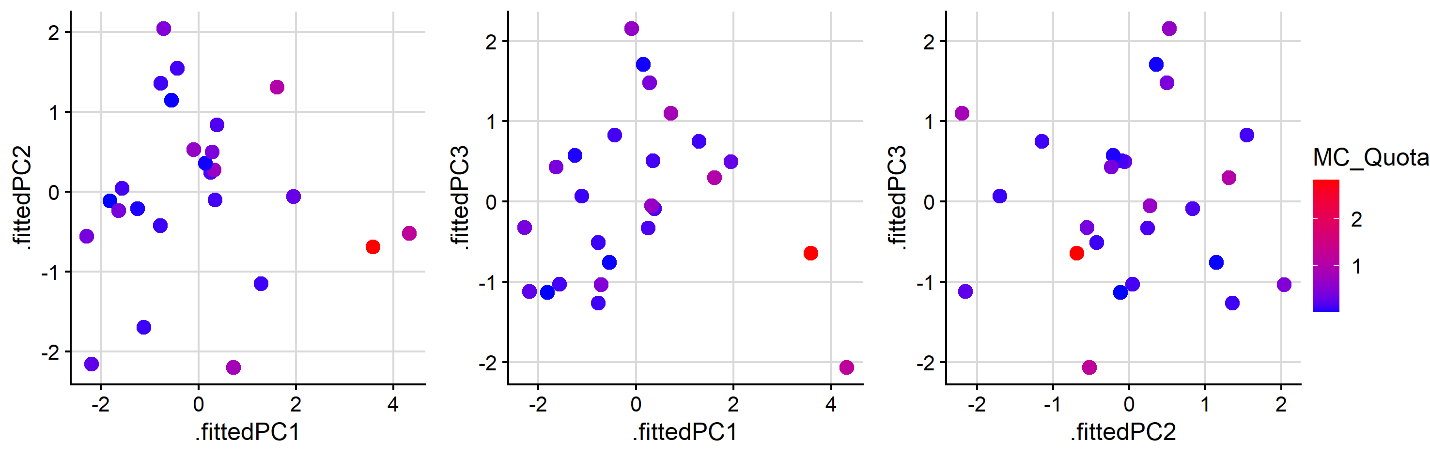


**S5d:** Computed correlations for each PC (red cells are above the 0.4 threshold and green cells are below the −0.4 threshold).

**S5e:** Biplots for combinations of PC1, PC2 and PC3 with predictor variables projected as vectors.


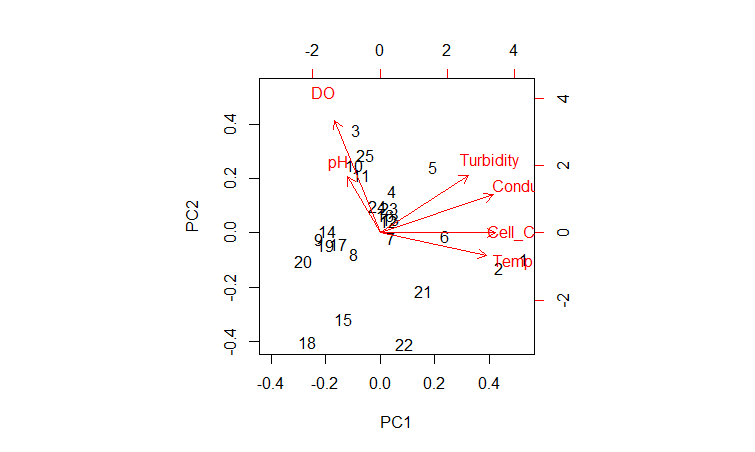

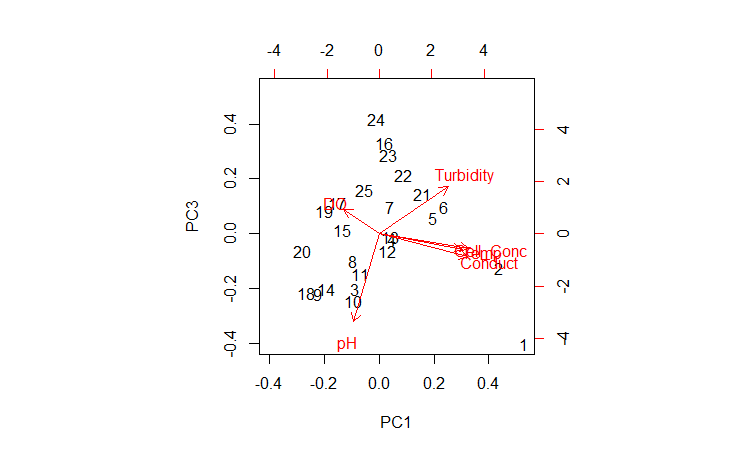

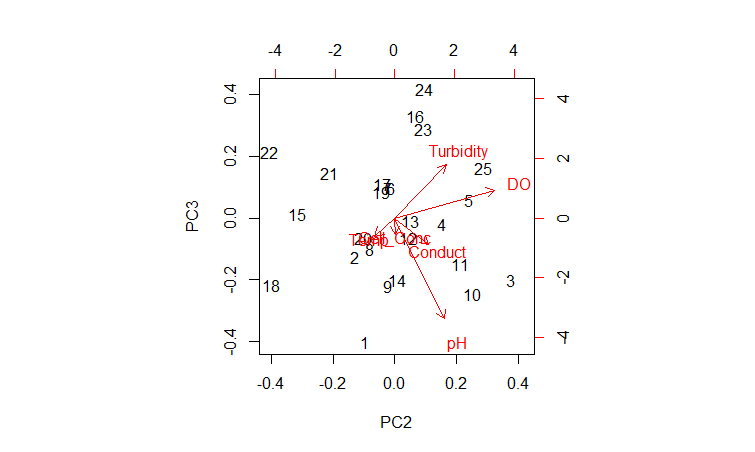


**S5f:** Pearson’s correlation matrix for predictor variables remaining in the analysis following PCA.


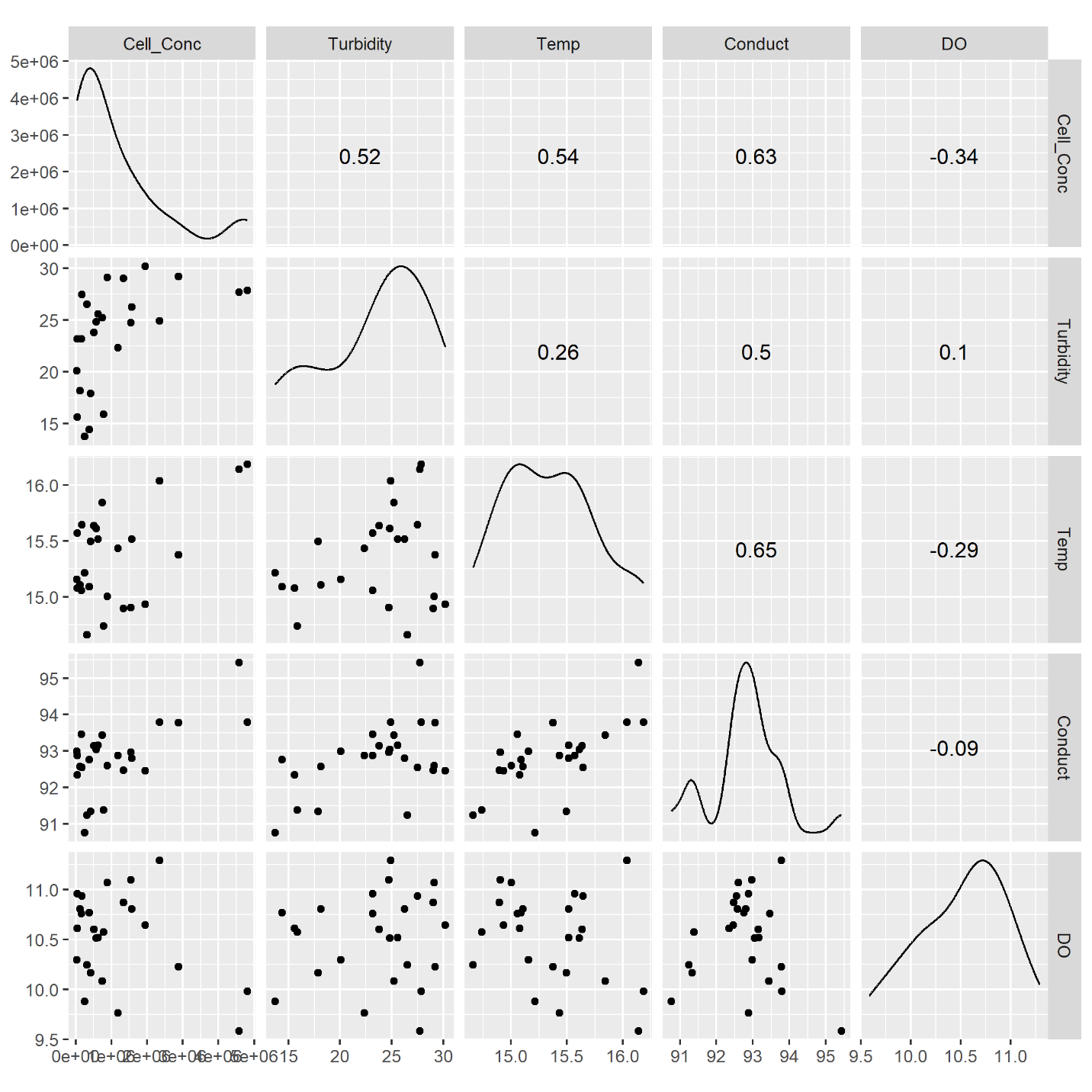


**S5g:** Variance inflation factors (VIFs) for predictor variables remaining following PCA and removal of highly-correlated variables.

**Supplementary Information S6:** Linear regression analysis of the relationship between microcystin quotas in the ‘bay study’ samples and *Microcystis* cell concentrations/physiochemical parameters.

**S6a:** Multiple linear regression model for prediction of microcystin quotas using all predictor variables remaining after PCA and data simplification.

**S6b:** Linear regression model for prediction of microcystin quotas using *Microcystis* cell concentration as the sole predictor variable.
